# Sharp Turning and Corner Turning: Comparison of Energy Expenditure, Gait Parameters, and Level of Fatigue among Community-Dwelling Elderly

**DOI:** 10.1155/2014/640321

**Published:** 2014-05-28

**Authors:** Maria Justine, Haidzir Manaf, Affeenddie Sulaiman, Shahir Razi, Hani Asilah Alias

**Affiliations:** ^1^Department of Physiotherapy, Faculty of Health Sciences, Universiti Teknologi MARA, Puncak Alam Campus, 42300 Puncak Alam, Selangor, Malaysia; ^2^Communities of Research (CoRe), Humanities and Quality of Life, Universiti Teknologi MARA, 40450 Shah Alam, Selangor Darul Ehsan, Malaysia

## Abstract

This study compares energy expenditure (EE), gait parameters (GP), and level of fatigue (LOF) between 5-minute walking with sharp turning (ST) and corner turning (CT). Data were obtained from 29 community-dwelling elderly (mean age, 62.7 ± 3.54 years). For 5 minutes, in ST task, participants walked on a 3-meter pathway with 2 cones placed at each end (180° turning), while in CT task, participants walked on a 6-meter pathway with 4 cones placed at 4 corners (90° turning). The physiological cost index, pedometer, and 10-point Modified Borg Dyspnoea Scale were used to measure EE (beats/min), GP (no of steps), and LOF, respectively. Data were analyzed by using independent *t*-tests. EE during ST (0.62 ± 0.21 beats/min) was significantly higher than CT (0.48 ± 0.17 beats/min) (*P* < 0.05). GP (434 ± 92.93 steps) and LOF (1.40 ± 1.11) in ST were found to be lower compared to GP (463 ± 92.18 steps) and LOF (1.54 ± 1.34) in CT (All, *P* > 0.05). Higher EE in ST could be due to the difficulty in changing to a 180° direction, which may involve agility and different turning strategies (step-turn or pivot-turn) to adjust the posture carefully. In CT, participants could choose a step-turn strategy to change to a 90° direction, which was less challenging to postural control.

## 1. Introduction

Age-related decline in sensory function and motor and neural processing, together with the common age-related diseases, results in balance impairment and increases the number of falls among older persons [1, 2]. Falls not only cause serious injuries, such as fractures and traumatic brain injuries, but also lead to accidental death. In addition, falls can lead to fear of falling, which results in progressive participation restriction [3]. This problem, in turn, can lead to several impairments, such as decreased muscle strength, balance, mobility, agility, and walking abilities as well as endurance. Hence, these outcomes can further result in frailty, loss of independence, and recurrent falls [4, 5].

Falls among independent community-dwelling older adults most often occur while walking on level or uneven surfaces [6]. Most falls occur at home, and the most common places are the bathrooms and stairs [7]. Turning or changing direction while walking is associated with falling incidences [8]. Falls and injuries during turning are eight times more likely to occur than during straight walking [9]. Turning while walking is a demanding task for older persons as it involves deceleration of forward motion, body rotation, and stepping out towards a new direction [10]. Staggering during turning, increased time, and the number of steps to complete turning are prominent characteristics of recurrent fallers [11].

Therefore, we surmise that, during turning, older persons may struggle to maintain their stability and to avoid falling. This study predicts that changing direction may cause the increased usage of energy due to the involvement of certain muscles. This statement is supported by previous studies, which claim that the instability experienced by older persons during walking is commonly associated with muscle weakness [12], delayed or reduced muscle activations, and inappropriately organized muscle response [13]. Another study reports that hip extension, trunk flexion, and step width are related to energy cost during walking [14]. However, intrinsic factors, such as energy expenditure, step frequency, and fitness, may influence the gait performance of older people.

Evidence shows that energy expenditure varies greatly among healthy older persons due to variations in physical activity [15]. In addition, gait parameters, such as step width and gait speed, are associated with energy cost in older adults with impaired mobility. In fact, energy expenditure is even associated with self-reported fatigue on a task requiring high levels of energy [16]. However, no study has investigated the effects of different turning strategies on energy expenditure, gait parameters, and level of fatigue during turning while walking.

Therefore, the objective of this study is to compare energy expenditure (EE), gait parameter (GP) (number of steps), and level of fatigue (LOF) between sharp turning (ST) and corner turning (CT) among community-dwelling older persons. We hypothesized that EE, GP, and LOF would differ with different turning strategies (ST and CT).

## 2. Methods

### 2.1. Participants

A convenient sample of 29 community-dwelling older persons (17 males, 12 females) from two different villages participated in this cross-sectional comparative study. The sample size of this study was calculated based on the previous studies [17, 18]. With 80% power and alpha at 5%, a sample size of 29 was sufficient to identify the differences in the findings. The inclusion criteria include (1) age 60 years and above, (2) ability to walk for at least 15 meters independently without a walking aid, (3) ability to follow simple orders and commands, (4) not being involved in regular exercise programs of more than 30 minutes per day for 3 times a week, and (5) being independent in their basic daily activities, such as cooking and bathing. Participants were excluded if they had self-reported neurologic disorders (e.g., Parkinson's disease and stroke), orthopedic conditions (e.g., joint deformities, osteoarthritis, and rheumatoid arthritis), and cardiorespiratory problems. In addition, participants with visual field defects and major hearing problems were excluded from this study. Prior to the procedure, participants were asked to sign informed consent forms that had been approved by the institutional ethics committee.

### 2.2. Procedure

Demographic data of the participants were recorded after the signing of the consent form. The items in the demographic form included current health status, age (years), height (m), weight (kg), body mass index (BMI) (kg/m^2^), and blood pressure (BP) (mmHg).

The physiological cost index (PCI) was used to determine the level of energy expenditure. The formula for calculating the PCI is adopted from a previous study [PCI (beats/min) = walking heart rate-resting rate (beats/min)/walking speed (m/min)] [18]. The reliability and validity of the PCI had been reported for healthy older persons while walking on two different tracks [19]. The intrarater (*r* = 0.73–*r* = 0.79) and interrater (*r* = 0.62–*r* = 0.66) reliability were acceptable between PCI scores from 20-meter and 12-meter tracks, respectively. In addition, in terms of walking duration, moderate ICC value (ICC = 0.57) and low level of agreement (LOA = 0.13–0.21) had been reported in a condition of 5 minutes of walking [20]. A portable pulse-oximeter was used to detect the heart rate throughout the test.

The number of steps was measured for both ST and CT by using a portable pedometer that was attached to the hips. A pedometer is a reliable tool in accurately detecting the number of steps taken by an older person [21]. In this study, a research assistant was also employed to calculate manually the number of steps recorded for completing all the tasks. The number of steps recorded via the pedometer and manual calculation were consistent, and thus, the number of steps from the pedometer was reported.

The level of fatigue was measured by using the 10-point Modified Borg Dyspnoea Scale before and after the test procedure and was based on the self-reported perception of the participant. The validity and reliability of Modified Borg Scale had been established in a previous study [22]. We also measured the total distance walked (TD) as well as the walking speed in both ST and CT tasks.

Participants were tested on two different occasions. The ST was conducted on the first day, which was followed by a 3-day rest aimed to minimize the training effects. On the fifth day, the CT was tested. Prior to testing, the tester demonstrated the procedure to the participants. On both testing sessions, the participants performed one round of practice to familiarize themselves with the test. Afterwards, the participants performed one trial for each of the tests on the respective day. Participants were asked to wear their regular footwear throughout the test procedure.

The procedure for the turning tasks was as follows.

**Figure 1:**
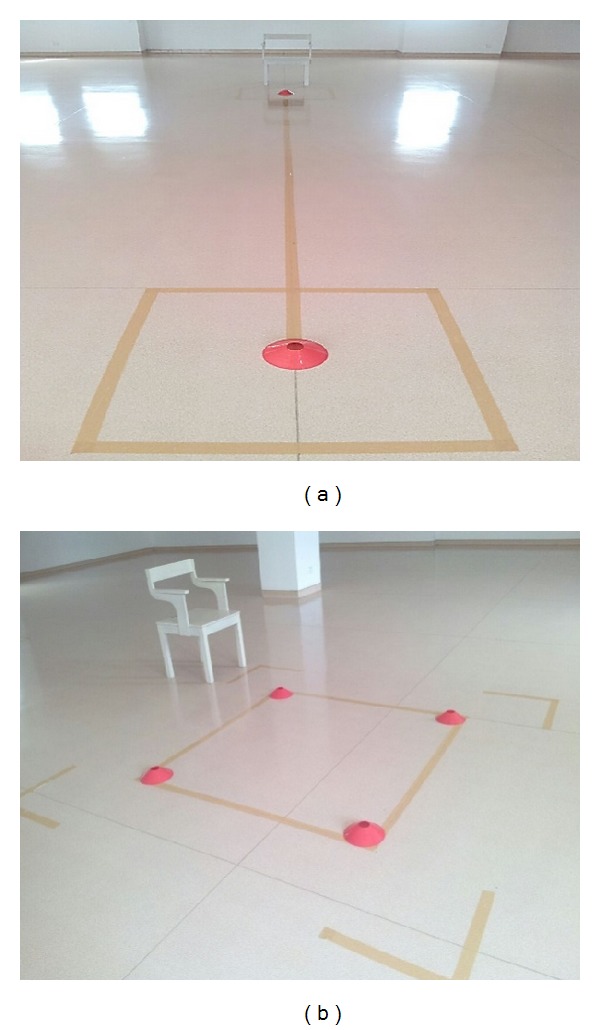
(a) and (b) show the tracks for ST and CT, respectively.

1. Sharp turning (ST): two cones were placed at two turning areas (beginning and end of a 3-meter distance) as shown in Figure 1(a). The turning area (1.0-meter by 1.0-meter) was marked on the floor to indicate the area around which participants were asked to turn. Prior to the test, pedometers and portable pulse-oximeters were attached to the participants. Baseline measurement from the Modified Dyspnoea Borg Scale was recorded. The participants were asked to walk at a comfortable pace, turn, and walk to the starting point again continuously for 5 minutes. The Modified Dyspnoea Borg Scale data were again recorded immediately once the task was completed.
2. Corner turning (CT): four cones were spaced at a distance of about 1.5 m from each other to form a square-shaped walking track as shown in Figure 1(b). Similar to the task mentioned above, participants were equipped with a pedometer and a portable pulse-oximeter. Baseline measurement from the Modified Dyspnoea Borg Scale was recorded. Participants then stood at the right distal corner of the track as marked on the floor. Then, they were asked to walk straight, perform a 90° left turn around the cones, and continuously perform the corner-turning task within 5 minutes. The Modified Dyspnoea Borg Scale result was recorded after the completion of the task.

### 2.3. Data Analysis

The data were analyzed by using SPSS statistical software version 18. Descriptive statistics were conducted to obtain the mean and the standard deviation for all outcome variables. The normality test showed that all data were normally distributed. The independent *t*-tests were performed to compare the significant differences in EE, GP, LOF, TD, and WS between the ST and CT tasks. The Pearson's correlation analysis was conducted between BMI and WS with EE, GP, and LOF in both ST and CT tasks to determine whether BMI and WS could be confounding factors in these variables. The level of significance of all statistical tests was assessed at *P* < 0.05.

## 3. Results

### 3.1. Demographics of Participants

A total of 29 participants completed the study procedure (17 males, 12 females).  Table 1 shows the age, height, weight, and BMI characteristics of all participants.

Table 2 indicates the results of the independent *t*-tests for comparisons of EE, GP, LOF, TD, and WS between ST and CT tasks. The EE in ST is significantly higher as compared to EE in CT (*P* = 0.008), and this finding may indicate that less energy was utilized during corner turning than sharp turning. Meanwhile, no significant differences were found in GP, LOF, TD, and WS between ST and CT tasks (both *P* > 0.05).

The analysis of correlation revealed that none of the variables (EE, GP, and LOF) were significantly correlated with BMI and walking speed (WS) in both ST and CT tasks (All, *P* > 0.05) (Table 3).

## 4. Discussion

The deterioration of gait performance (number of steps and time taken) during turning while walking has been reported among older persons [22–25]. However, little is known about the effects of two different turning strategies on EE, GP, and LOF in older persons. The understanding of fall occurrence during turning strategies while walking a short course length is limited. As such, in this study, we implemented a 3-meter and a 1.5-meter course length to represent large and small space areas in a home-based setting, respectively. Recent studies revealed that 66.8% of falls occurred inside the home [26], with falls in the bathroom resulting in injuries that were 2.5 times higher compared to falls in the living room.

We argue for the importance of studying EE, which varies greatly among healthy older persons due to variations in physical activity as reported in an earlier study. Therefore, we hypothesized that EE, GP, and LOF differed between two turning strategies (ST and CT). Our results indicated that, first, EE deteriorated more during ST than CT, and, second, ST and CT did not lead to differential effects in GP and LOF.

### 4.1. Energy Expenditure

This study revealed higher EE during the ST task compared to the CT task. This finding might have been caused by the different strategies participants used to maintain balance and equilibrium during ST. To complete a turn, a clear temporal sequence in the initiation of axial segment reorientation is needed. The steering task is first initiated by the head yaw direction, followed by the trunk, and finally the feet in a craniocaudal sequence [27]. However, during ST, older persons may exhibit less movement of head on trunk rotation in the presence of age-related decreases in cervical spine rotation and low balance confidence to change to a 180° walking direction. This reduced head on trunk rotation was partly compensated by increased trunk movement on pelvis rotation. Contradicting the CT strategies, a previous study showed that older persons demonstrated an en-bloc method of segmental reorientation to simplify the control of the movement and to minimize the risk of imbalance during 90° turning [28]. In addition, a higher amount of energy expenditure is needed to contract the muscles of the lower limb, while, at the same time, the abdominal muscle contracts and cooperates to sustain the stability of the body [29]. By contrast, the increased requirement of energy in achieving a task goal is found to be associated with bodily controlled movement [30]. However, the required energy is different when compared to the energy exerted during corner turning. The reduction in the angle of turning helps to maintain sufficient space for maintaining a proper base of support. Therefore, less energy is needed to maintain balance and stability. Increased muscle activation requires more metabolic energy cost of performance [31]. Therefore, ST requires more energy even though the course length was longer than that of CT. During this maneuver, a bodily sequence strategy to maintain balance and a greater number of muscles may need to be activated to perform the turning at a sharp angle. Therefore, the level of EE may vary across different types of activities. This finding supports the conclusions of a previous study [15].

### 4.2. Gait Parameters

The second objective of this study is to compare the number of steps between ST and CT. Contrary to our expectation, we found that more steps were employed during CT, even though the differences in the number of steps were not significant (*P* > 0.05). An earlier study reported that a turning activity might alter the GP while walking [32]. Despite that finding, another study found that the turning activities of young adults and older people are affected by the spatiotemporal measure during walking, which includes step frequency. We believe that this result could be due to the ability of the older persons in this study to overcome the instability from the forward momentum during turning. This finding might have resulted from the difficulty in changing a 180° direction of turning during ST, which may involve agility and different turning strategies (step-turn or pivot-turn) to adjust the posture carefully.

In CT, the participants most likely chose the spin-turn or step-out strategy when they were required to change to a 90° direction, which was less challenging to postural control. Consistent with our findings, spin-turns were more frequent among older persons compared to step turns [33]. Besides that, a decrease in step frequency during ST compared to the step frequency in CT was also reported in this study. This observation could be the result of lesser total distance and lower walking speed achieved in ST compared to CT. During ST, longer anticipatory postural adjustment (APA) may be needed to compensate the body to maintain stability and to avoid loss of balance [28]. The current health status of the participants might also assist them in maintaining good balance while performing the test. Most of the participants are rural older persons who are physically active in carrying out their functional daily activities. Therefore, they were able to adapt to the force of inertia during both ST and CT.

Another plausible explanation is that the participants of this study might increase step frequency only when changing direction. This finding was also supported by a previous study that evaluated the specific measurement of kinematic and kinetic of turning. The study reported an increased amount of knee and plantar flexion, which indicated an increased swing phase of gait [34] and, thus, produced a higher number of step frequencies. Other similar results of increases in the number of steps in turning activity have been found [23]. The study suggested that more turning maneuvers required large portions of steps taken compared to straight walking. This observation supports our finding why CT presented a higher number of steps as the procedure involved four corners for turning.

### 4.3. Level of Fatigue

The third objective of the study is to compare the LOF. We found no significant differences on the LOF between ST and CT. Hence, we concluded that both turning strategies that were completed within 5 minutes were not exhaustive enough for the healthy older persons, even though the statistical results showed a slightly higher mean of fatigue during the CT task. The reason underlying this finding is that the spin-turn strategy during CT requires greater biomechanical cost that comes from greater demands in the ankle inventor and hip abductor activities of the supporting leg [33]. The changes in the levels of fatigue before and after walking and turning can also be related to the higher energy exerted during the performance of ST and CT.

A study conducted among multiple sclerosis patients corresponded to our study result [35]. Participation in physical activity is influenced by fatigue and thermosensitivity characteristic, which is a neurological condition that worsens when the body temperature increases. Similarly, their study showed that more turning maneuvers performed by the participants would decrease the level of endurance due to fatigue. Another study suggested that older women who suffered from breast cancer and were reported to have higher levels of physical activity reported less fatigue [36]. This result corroborated the findings of our research. A study among older persons over 65 years old found that participants who consumed high amount of calories coupled with low levels of physical activity showed significant fatigue symptoms when performing high intensity exercises [37]. This observation may explain why the repetitive changing direction during walking requires more energy exertion and causes rapid fatigue among our participants.

### 4.4. Study Limitation

A major limitation to this study concerns the characteristics of the study participants. The participants for this study were recruited from a community dwelling. They are healthy and independent in their daily activities. They have the ability to go out of their houses to attend social functions around the community. These activities normally require them to walk for more than 5 minutes, and this routine may control the effect of fatigue as indicated in the test procedure of this study. Therefore, we suggest that this study be repeated among frail older persons and individuals with chronic illnesses. A longer duration of the test procedure may be needed to determine the exact moment when an older person experiences the onset of fatigue. This situation may help to identify the consequences of fatigue to the body, such as postural control. In addition, other factors, such as base of support (BOS) and step width, during walking may need to be measured to determine whether these factors contribute to EE and LOF. Another potential weakness of this study can be attributed to the unequal number of male and female participants. Physiologically, male participants have a higher functional capacity compared to females. Besides, the counterbalanced effects of ST and CT were not clearly addressed in this study. We first tested the ST and followed by the CT. Learning or adaptation effects might have occurred between subjects and might have contributed to the bias in the overall performance of the measured variables.

## 5. Conclusion and Recommendation

In conclusion, in ST, EE was significantly higher compared to CT. However, GP and LOF were lower in ST although the result did not reach a significant level. We believe that the higher EE in ST is affected by the difficulty in changing to a 180° direction as the movement may involve agility and different turning strategies (step-turn or pivot-turn) to adjust the posture carefully. In CT, participants may choose a spin-turn strategy to change a 90° direction, which is less challenging to postural control.

In the rehabilitation of older persons, a proper design of exercises should include various activities that reflect the need to enhance their functional daily activities and their safety. People have always used inappropriate methods or strategies to handle risky movements, such as outdoor locomotion, climbing stairs, and walking in uneven, narrowed, and winding pathways. Therefore, we recommend that gait rehabilitation for older adults, especially among those with poor functional capacity, should include challenges, such as ST, CT, and so forth. In addition, we suggest that exercise designs for older people should include innovation in turning activities to promote economic energy cost and safe turning strategies.

## Figures and Tables

**Table 1:** Characteristics of participants.

Characteristics (*N* = 29)	Value
Age, year, mean (±SD); (Range)	62.7 (±3.54); (60–72)
Female, *n* (%)	12 (41)
Male, *n* (%)	17 (59)
Height, m, mean (±SD); (Range)	1.6 (±0.078); (1.43–1.72)
Weight, kg, mean (±SD); (Range)	66.76 (±11.96); (42–90.7)
BMI, kg/m^2^, mean (±SD); (range)	26.17 (±4.45); (17.48–34.48)

**Table 2:** Comparisons of EE, GP, LOF, TD, and WS between ST and CT tasks.

Variables	ST (*n* = 29) Mean (SD)	CT (*n* = 29) Mean (SD)	Mean difference (95% CI)	*t*-stats (df)	*P* value
EE	0.620 (0.21)	0.483 (0.17)	0.137 (0.036, 0.237)	2.73 (56)	0.008*
GP (number of steps)	434 (94.56)	463 (93.81)	−29 (−78.446, 20.652)	−1.168 (56)	0.25
LOF	1.397 (1.11)	1.535 (1.34)	−0.138 (−0.787, 0.511)	−0.426 (56)	0.67
TD	138.71 (32.98)	146.84 (34.45)	−8 (−25.8758, 9.6048)	−0.919 (56)	0.36
WS	27.74 (6.60)	29.37 (6.90)	−1.6 (−5.1752, 1.9210)	−0.919 (56)	0.36

ST: sharp turning; CT: corner turning; EE: energy expenditure; GP: gait parameters, LOF: level of fatigue; TD: total distance; WS: walking speed. *Significant at *P* < 0.05.

**Table 3:** Correlations between BMI and walking speed with EE, GP, and LOF in both ST and CT.

Variables	BMI *r* (*P* value)	Walking speed *r* (*P* value)
ST		
EE	0.228 (0.235)	−0.256 (0.180)
GP	0.261 (0.172)	0.359 (0.056)
LOF	0.187 (0.331)	0.168 (0.385)

CT		
EE	−0.120 (0.535)	0.088 (0.648)
GP	0.280 (0.142)	0.358 (0.056)
LOF	0.102 (0.597)	0.115 (0.553)

ST: sharp turning; CT: corner turning,; EE: energy expenditure; GP: gait parameters; LOF: level of fatigue; TD: total distance; WS: walking speed.

## References

[1] Maki BE, McIlroy WE (2006). Control of rapid limb movements for balance recovery: age-related changes and implications for fall prevention. *Age and Ageing*.

[2] Sturnieks DL, St George R, Lord SR (2008). Balance disorders in the elderly. *Neurophysiologie Clinique*.

[3] Allison LK, Painter JA, Emory A, Whitehurst P, Raby A (2013). Participation restriction, not fear of falling, predicts actual balance and mobility abilities in rural community-dwelling older adults. *Journal of Geriatric Physical Therapy*.

[4] Sharaf AY, Ibrahim HS (2008). Physical and psychosocial correlates of fear of falling among older adults in assisted living facilities. *Journal of Gerontological Nursing*.

[5] Visschedijk J, Achterberg W, van Balen R, Hertogh C (2010). Fear of falling after hip fracture: a systematic review of measurement instruments, prevalence, interventions, and related factors. *Journal of the American Geriatrics Society*.

[6] Berg WP, Alessio HM, Mills EM, Tong C (1997). Circumstances and consequences of falls in independent community-dwelling older adults. *Age and Ageing*.

[7] Sazlina S, Krishnan R, Shamsul A, Zaiton A, Visvanathan R Prevalence of falls among older people attending a primary care in Kuala Lumpur, Malaysia. *Journal of Community Health*.

[8] Patla AE, Frank JS, Winter DA (1992). Balance control in the elderly: implications for clinical assessment and rehabilitation. *Canadian Journal of Public Health*.

[9] Cumming RG, Klineberg RJ (1994). Fall frequency and characteristics and the risk of hip fractures. *Journal of the American Geriatrics Society*.

[10] Hase K, Stein RB (1999). Turning strategies during human walking. *Journal of Neurophysiology*.

[11] Tinetti ME, Williams TF, Mayewski R (1986). Fall risk index for elderly patients based on number of chronic disabilities. *American Journal of Medicine*.

[12] Wittink H, Engelbert R, Takken T (2011). The dangers of inactivity; exercise and inactivity physiology for the manual therapist. *Manual Therapy*.

[13] Lin S, Woollacott MH (2002). Postural muscle responses following changing balance threats in young, stable older, and unstable older adults. *Journal of Motor Behavior*.

[14] Wert DM, Brach J, Perera S, VanSwearingen JM (2010). Gait biomechanics, spatial and temporal characteristics, and the energy cost of walking in older adults with impaired mobility. *Physical Therapy*.

[15] Goran MI, Poehlman ET (1992). Total energy expenditure and energy requirements in healthy elderly persons. *Metabolism: Clinical and Experimental*.

[16] Buchowski MS, Simmons SF, Whitaker LE (2013). Fatigability as a function of physical activity energy expenditure in older adults. *Age*.

[17] Danielsson A, Sunnerhagen KS (2004). Energy expenditure in stroke subjects walking with a carbon composite ankle foot orthosis. *Journal of Rehabilitation Medicine*.

[18] Peebles KC, Woodman-Aldridge AD, Skinner MA (2003). The physiological cost index in elderly subjects during treadmill and floor walking. *The New Zealand Journal of Physiotherapy*.

[19] Graham RC, Smith NM, White CM (2005). The reliability and validity of the physiological cost index in healthy subjects while walking on 2 different tracks. *Archives of Physical Medicine and Rehabilitation*.

[20] Danielsson A, Willén C, Sunnerhagen KS (2007). Measurement of energy cost by the physiological cost index in walking after stroke. *Archives of Physical Medicine and Rehabilitation*.

[21] Dijkstra B, Zijlstra W, Scherder E, Kamsma Y (2008). Detection of walking periods and number of steps in older adults and patients with Parkinson’s disease: accuracy of a pedometer and an accelerometry-based method. *Age and Ageing*.

[22] Tanaka K, Akechi T, Okuyama T, Nishiwaki Y, Uchitomi Y (2000). Development and validation of the Cancer Dyspnoea Scale: a multidimensional, brief, self-rating scale. *British Journal of Cancer*.

[23] Glaister BC, Bernatz GC, Klute GK, Orendurff MS (2007). Video task analysis of turning during activities of daily living. *Gait and Posture*.

[24] Meinhart-Shibata P, Kramer M, Ashton-Miller JA, Persad C (2005). Kinematic analyses of the 180° standing turn: effects of age on strategies adopted by healthy young and older women. *Gait and Posture*.

[25] Thigpen MT, Light KE, Creel GL, Flynn SM (2000). Turning difficulty characteristics of adults aged 65 years or older. *Physical Therapy*.

[26] Stevens JA, Mahoney JE, Ehrenreich H (2014). Circumstances and outcomes of falls among high risk community-dwelling older adults. *Injury Epidemiology*.

[27] Hollands MA, Ziavra NV, Bronstein AM (2004). A new paradigm to investigate the roles of head and eye movements in the coordination of whole-body movements. *Experimental Brain Research*.

[28] Wright RL, Peters DM, Robinson PD, Sitch AJ, Watt TN, Hollands MA (2012). Differences in axial segment reorientation during standing turns predict multiple falls in older adults. *Gait and Posture*.

[29] Sparrow WA, Newell KM (1998). Metabolic energy expenditure and the regulation of movement economy. *Psychonomic Bulletin and Review*.

[30] Sparrow WA, Hughes KM, Russell AP, le Rossignol PF (1999). Effects of practice and preferred rate on perceived exertion, metabolic variables and movement control. *Human Movement Science*.

[31] Lay BS, Sparrow WA, Hughes KM, O’Dwyer NJ (2002). Practice effects on coordination and control, metabolic energy expenditure, and muscle activation. *Human Movement Science*.

[32] Shkuratova N, Morris ME, Huxham F (2004). Effects of age on balance control during walking. *Archives of Physical Medicine and Rehabilitation*.

[33] Akram SB, Frank JS, Chenouri S (2010). Turning behavior in healthy older adults: is there a preference for step versus spin turns?. *Gait and Posture*.

[34] Orendurff MS, Segal AD, Berge JS, Flick KC, Spanier D, Klute GK (2006). The kinematics and kinetics of turning: limb asymmetries associated with walking a circular path. *Gait and Posture*.

[35] Fjeldstad C, Brittain DR, Fjeldstad AS, Pardo G (2010). Fatigue and thermo sensitivity affect physical activity in multiple sclerosis. *Journal of Applied Research*.

[36] Winters-Stone KM, Bennett JA, Nail L, Schwartz A (2008). Strength, physical activity, and age predict fatigue in older breast cancer survivors. *Oncology Nursing Forum*.

[37] Soyuer F, Şenol V (2011). Fatigue and physical activity levels of 65 and over older people living in rest home. *International Journal of Gerontology*.

